# A Rare Case of Giant Esophageal Leiomyoma in an Adult Male Presenting With Right Upper Quadrant Pain and Mild Dysphagia

**DOI:** 10.7759/cureus.89349

**Published:** 2025-08-04

**Authors:** Seeduwa Bandara, Clifford Pang, Katherine Pang, Ronald Tsao

**Affiliations:** 1 Medicine and Pediatrics, University of California Los Angeles David Geffen School of Medicine, Los Angeles, USA; 2 Family Medicine, University of California Los Angeles David Geffen School of Medicine, Los Angeles, USA

**Keywords:** esophagus, fna, leiomyomas, mesenchymal tumors, muscle sparing thoracotomy

## Abstract

Leiomyomas are the most common benign mesenchymal tumors of the esophagus, and they account for nearly two-thirds of benign esophageal neoplasms. The leiomyomas of the esophagus present with numerous nonspecific symptoms and signs, including dysphagia, shortness of breath, anorexia, weight loss, chronic cough, and bowel obstruction. The patient in this case report presented with moderate to severe right upper quadrant pain and mild dysphagia, which initially prompted evaluation for hepatobiliary pathology. However, the subsequent imaging incidentally showed a tumor surrounding the middle and lower third esophagus. Endoscopic findings identified a large submucosal mass, and the fine needle aspiration (FNA) results confirmed leiomyoma, excluding leiomyosarcoma. Due to the size of the tumor, the patient underwent surgery with muscle-sparing thoracotomy. His recovery was uneventful, and no complications were reported during follow-up visits.

## Introduction

Esophageal leiomyomas are slow-growing neoplasms that vary in size and can present with a wide range of symptoms. Tumors that grow larger than 10 cm in diameter are considered “giant leiomyomas” [1]. CT imaging is useful in evaluating these tumors and can help distinguish between benign and malignant lesions. Surgical resection remains the treatment of choice in symptomatic leiomyomas [2-3]. Open resection, video-assisted thoracoscopic surgery (VATS)-guided enucleation, thoracoscopic enucleation, robot-assisted thoracoscopic resection, transthoracic resection, and endoscopic resection are the surgical techniques that are widely used, depending on the location, extension, and size of the tumor [4].

## Case presentation

**Figure 1:**
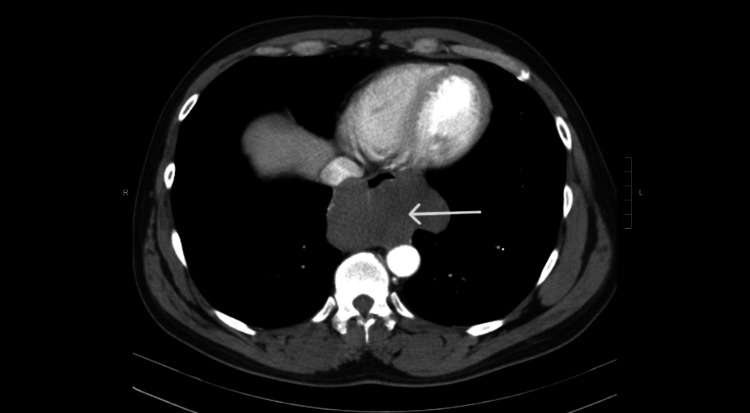
Contrast-enhanced CT abdomen demonstrating esophageal leiomyoma (white arrow) CT: computed tomography

A 39-year-old male presented with right upper quadrant pain that radiated to the right lower chest and was associated with acid reflux for five months. The patient had used over-the-counter anti-acid medications without improvement. He reported mild dysphagia, but denied loss of appetite, anorexia, weight loss, chest pain, shortness of breath, fever, or chills. There was no history of diarrhea or constipation. He occasionally drank alcohol and was never a smoker. His family and social history were noncontributory. On examination, the vital signs were within normal limits. The lung exam was also normal. Auscultation of the precordium showed normal heart sounds with no murmurs. Normal bowel sounds were present during the abdominal exam without any bruits. The palpation of the abdomen revealed tenderness over the right upper quadrant without organomegaly. Basic laboratory investigations, including renal and hepatic profiles, were within the reference range. Abdominal ultrasonography was normal. A subsequent chest radiograph showed a mass in the chest cavity. This was followed up with a contrast-enhanced chest CT that showed an esophageal mass of the lower two-thirds measuring 10 x 8 x 6 cm without any signs of metastatic disease (Figure 1).

**Figure 2:**
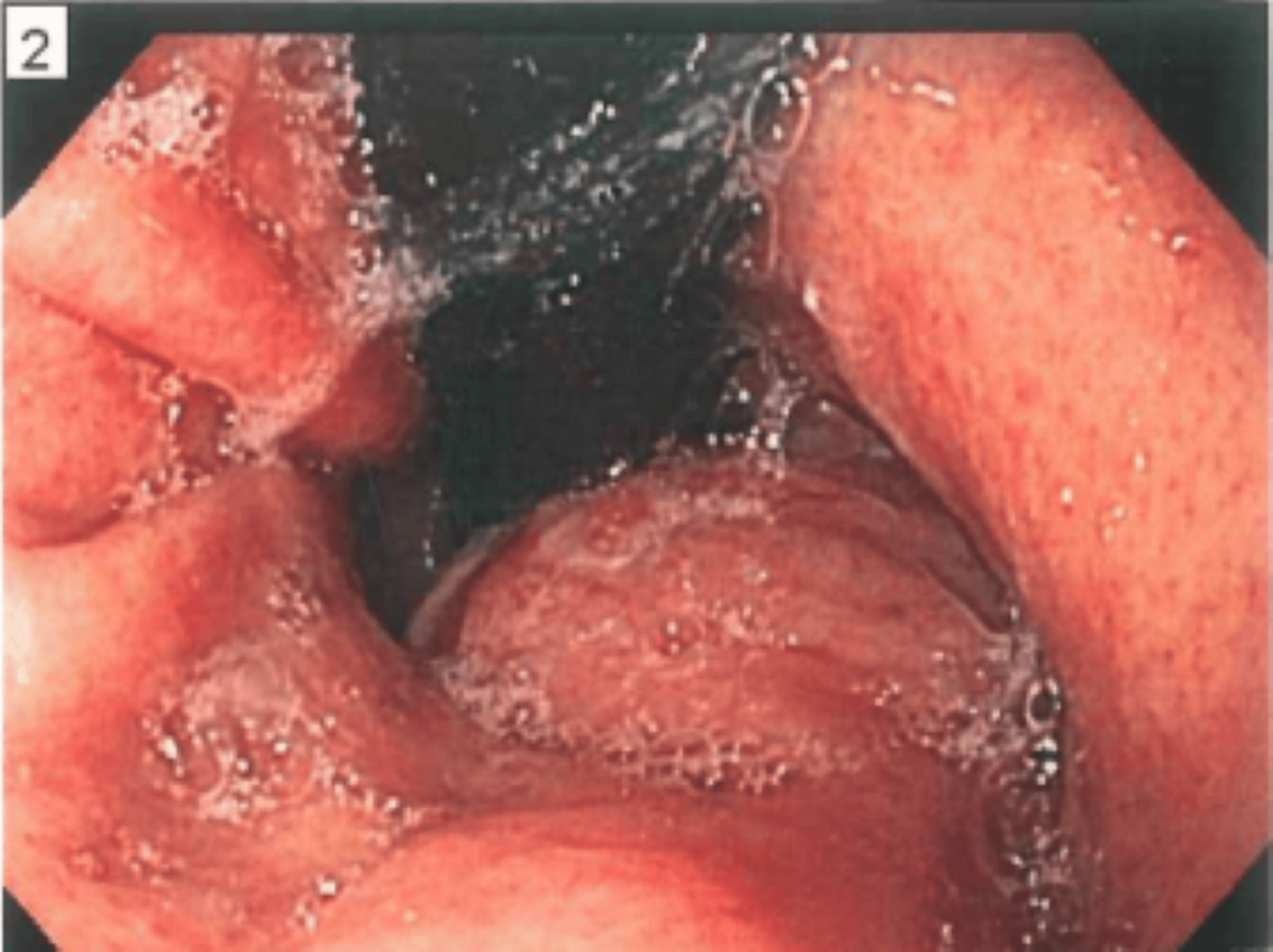
Endoscopy revealing submucosal leiomyoma

Endoscopic evaluation revealed an 82 x 76 mm partially obstructing oval intramural tumor extending from mid to the distal esophagus (Figure 2). Fine needle aspiration (FNA) pathology showed positivity for smooth muscle actin and desmin, and was negative for malignant markers such as S100, CD 117 and CD34 on immunohistochemistry [5-6]. The patient was scheduled for VATS, which was converted to a left posterolateral muscle-sparing thoracotomy due to the extensive nature of the almost two-pound mass. The histopathological evaluation of the respective mass confirmed the FNA findings. The patient had an uneventful postoperative period. He was able to tolerate clear liquids and gradually advanced to a soft diet in the next six weeks. His thoracostomy tubes were removed, and he was discharged on postoperative day four. He had an uneventful recovery and was found to be doing well at his six-week follow-up visit.

## Discussion

We described a rare case of a leiomyoma of the lower two-thirds of the esophagus that presented with abdominal pain. The initial presentation led the providers to focus on the hepatobiliary system, but subsequent imaging studies supported the eventual diagnosis. Esophageal leiomyomas manifest symptoms with their growth mainly due to compression of surrounding structures. In our case, it was notable that the initial symptoms manifested away from the lesion’s primary site, possibly due to nerve compression. CT imaging plays a vital role in diagnosing and characterizing esophageal tumors. CT features such as size, contour, enhancing pattern, mesenteric fat infiltration, calcification, ulceration, and direct invasion, and regional lymphadenopathy help in determining the nature of the tumor activity [2-3]. In this case, the absence of direct invasion and distant metastasis helped differentiate the leiomyoma from leiomyosarcoma.

Given the recognized heterogeneity of large tumors, comprehensive histopathological evaluation was deemed essential [5-6]. Accordingly, both histopathological and histochemical analyses were performed preoperatively and postoperatively following tumor resection. The preferred surgical treatment for leiomyoma is enucleation via thoracotomy or thoracoscopy. Minimally invasive procedures are now gaining more attention due to reduced postoperative hospital stay and a faster recovery period. However, open surgery is required for giant leiomyomas that need esophageal resection and reconstruction [7].

## Conclusions

Esophageal leiomyomas may present with various nonspecific clinical symptoms. Therefore, it is essential to maintain a high index of suspicion and perform a thorough clinical evaluation. CT imaging plays a pivotal role in establishing an accurate diagnosis while understanding the tumor behavior. Endoscopic ultrasound-guided FNA further establishes the diagnosis. The size and location of the leiomyoma can influence surgical planning. There are various surgical approaches being studied for surgical enucleation. Newer minimally invasive techniques have become more popular due to better post-surgical outcomes, such as shorter length of hospital stay and shorter recovery time.
